# Commentary: MARCH8 Inhibits HIV-1 Infection by Reducing Virion Incorporation of Envelope Glycoproteins

**DOI:** 10.3389/fmicb.2016.00254

**Published:** 2016-02-24

**Authors:** Mikako Fujita

**Affiliations:** Research Institute for Drug Discovery, School of Pharmacy, Kumamoto UniversityKumamoto, Japan

**Keywords:** MARCH8, SAMHD1, macrophage, HIV, reservoir

Recently, Kenzo Tokunaga's group reported a novel restriction factor against HIV, MARCH8, which is highly expressed in terminally differentiated myeloid cells such as macrophages. Virus infection in macrophages was first observed in HIV-infected patients in the mid-1980s (Gyorkey et al., 1985; Ho et al., 1986; Koenig et al., 1986). Three decades have passed since then; however, the role of HIV-infected macrophages in AIDS pathogenesis remains controversial. Here, some potential implications of Tokunaga et al.'s study on this controversy will be addressed.

SIV-infected rhesus monkeys are a good model for investigating the role of HIV-infected macrophages because their pathology resembles the slow progression of AIDS in humans. A comparative study of rhesus macaques infected with T cell-tropic SIVmac239 (Kestler et al., 1990) or with macrophage-tropic SIVmac316 (Mori et al., 1992), which carries nine mutations compared with SIVmac239 (Johnson et al., 2003) found that SIVmac316 replicates with the simian body as well as SIVmac239 just after inoculation. However, SIVmac316 induces a slower disease progression than SIVmac239, demonstrating that the contribution of virus-infected macrophages to pathogenesis is smaller than that of virus-infected T cells.

Studies have also used an SIV that lacks expression of its accessory protein, Vpx, which is critical for SIV/HIV-2 replication in macrophages and resting T lymphocytes and is also important in activated T lymphocytes (Fujita et al., 2010, 2012; Baldauf et al., 2012). Rhesus macaques infected with a *vpx*-deleted SIVmac239 eventually died after a slower disease progression than that of animals infected with wild-type SIVmac239 (Westmoreland et al., 2014). In monkeys infected with *vpx*-deleted SIVmac239, minimal macrophage infection was detected, even though infected macrophages were observed following wild-type SIV infection.

Furthermore, there was a recent study of rhesus macaques infected with SIVmac239 or SIVmac316 mutants, both of which had mutations in Vpx inhibiting the ability of this protein to confer infectivity. The viruses that recovered their replication ability in this study only appeared in the animals infected with the T cell-tropic SIVmac239, demonstrating the lower importance of virus replication in macrophages than that in T cells (Shingai et al., 2015). Based on these results, it is likely that HIV has difficulty replicating in macrophages *in vivo* and that HIV-infected macrophages play a minimal role in progressing the general symptom of AIDS.

Although HIV-infected macrophages are not critical for disease progression, their role in HIV infection may be to serve as an HIV reservoir in the body, acting as an obstacle to HIV eradication by antiretroviral therapy (ART). The existence of an HIV reservoir has been postulated since just after the establishment of ART (Chun and Fauci, 1999), and although a 2001 report suggested that it might be composed of macrophages (Igarashi et al., 2001), until recently (Churchill et al., 2016) it was generally believed to be composed of memory CD4^+^ T cells, partially because it is difficult to elucidate which cell type(s) constitute the reservoir by using patients or through laboratory experiments. In support of the hypothesis that macrophages are the HIV reservoir, HIV-infected macrophages were observed in HIV-infected patients with undetectable plasma viral loads (Cribbs et al., 2015). Even taking into account that macrophages are resistant to HIV replication, macrophages may serve as part of long-lived HIV reservoir.

Great progress into understanding HIV-resistance in macrophages, specifically the discovery of two host restriction factors in macrophages, has recently been made. One of these restriction factors is SAMHD1 (Hrecka et al., 2011; Laguette et al., 2011). This protein was found as a target protein of Vpx, and it reduces reverse transcription (RT) products. Investigations into the function of SAMHD1 initially focused on its dNTPase activity (Goldstone et al., 2011; Powell et al., 2011), reducing dNTP pools, materials of genomic cDNA (Kim et al., 2012; Lahouassa et al., 2012). However, it was later proposed that SAMHD1 uses its RNase activity to degrade HIV RNA before reverse transcription (Beloglazova et al., 2013; Ryoo et al., 2014). It is presently unclear if one of these or both are responsible for the activity of SAMHD1 (Ballana and Esté, 2015). Interestingly, the HIV-2 Vpx protein is able to degrade SAMHD1 (Hrecka et al., 2011; Laguette et al., 2011), while HIV-1 lacks a special protein to combat SAMHD1. Although the reverse transcriptase of HIV-1 is more efficient than that of HIV-2 (Lenzi et al., 2015), the artificial incorporation of Vpx into HIV-1 virions dramatically increases their infectivity in macrophages (Goujon et al., 2008), showing that HIV-1 does not sufficiently overcome the function of SAMHD1.

Another recently discovered host factor in macrophages is membrane-associated RING-CH8 (MARCH8) (Tada et al., 2015). This protein has been known to downregulate various transmembrane proteins. As with many great scientific discoveries, the identification of MARCH8 as a macrophage host factor began with a serendipitous finding. Tada et al. initially noticed that MARCH8-expressing lentiviral vectors had a low infectivity and later found that a large amount of MARCH8 is specifically expressed in terminally differentiated myeloid cells, macrophages, and dendritic cells. MARCH8 was demonstrated to drastically reduce HIV-1 virion incorporation of envelope glycoproteins and inhibit its infectivity. The same inhibitory effect was observed in virions containing envelope proteins from HIV-2, SIV, MLV, or VSV. MARCH8 was suggested to interact with HIV-1 Env, leading to its downregulation from surface of producer cells. Interestingly, neither HIV-1 Vpr, Vpu nor Nef have detectable anti-MARCH8 activity, suggesting that HIV-1 lacks a mechanism to directly combat the effects of MARCH8.

HIV, particularly HIV-1, may have evolved a way of taking advantage of the effects of host restriction proteins such as SAMHD1 and MARCH8 (Figure 1). The synergistic suppression of infectivity by these factors and other effects likely leads to a mild amount of HIV replication in macrophages, causing minimal cellular damage. Furthermore, virus could escape from host immune system. These permit virus survival. The long life of these cells allows them to serve as viral reservoirs, present even in patients with undetectable plasma viral loads after receiving ART. Future studies should aim to devise ways of targeting the macrophage reservoir cells to fully eliminate HIV.

**Figure 1:**
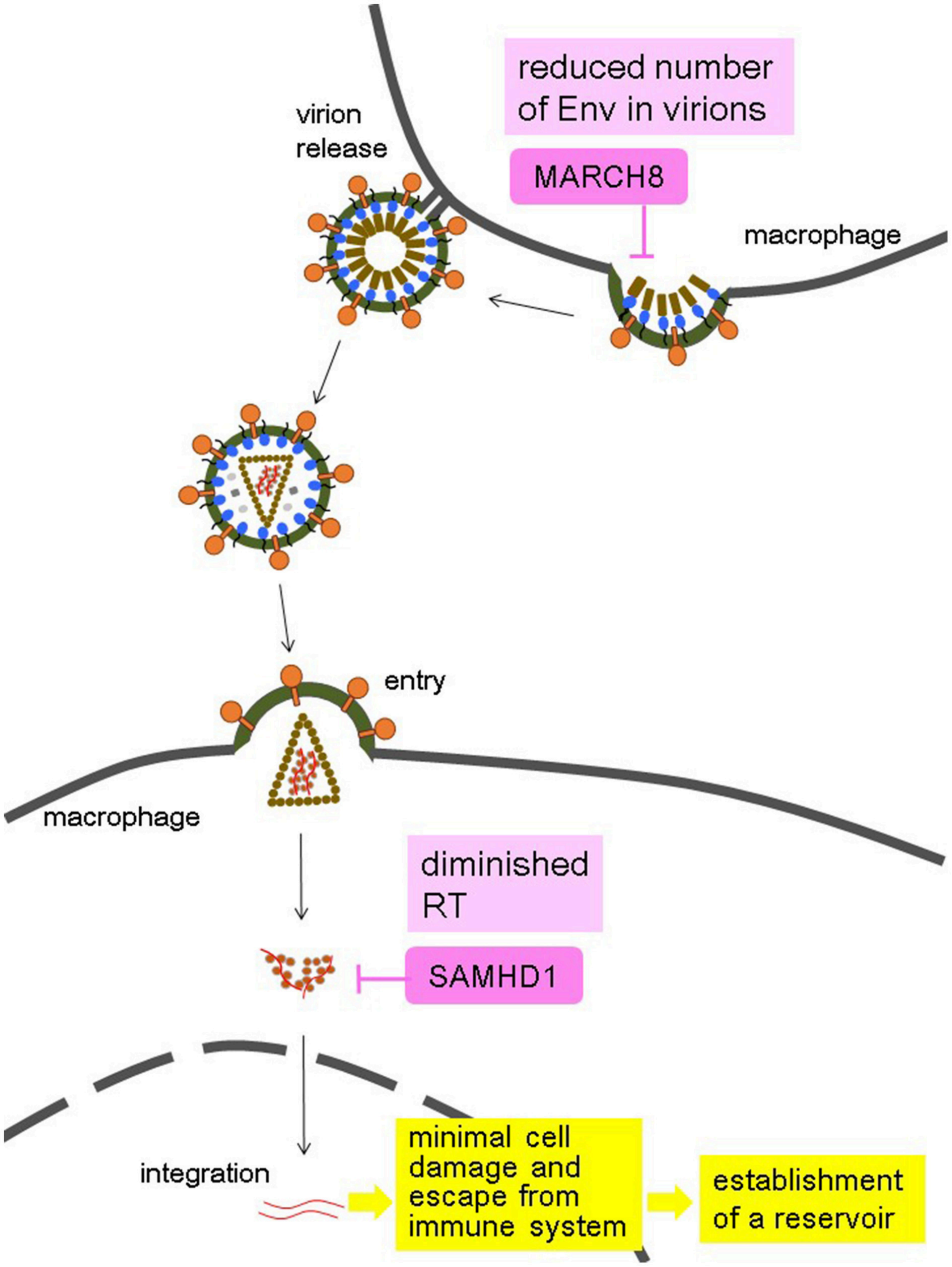
**Hypothesis of the HIV-1 strategy to use macrophages as a reservoir by taking advantage of the synergistic effects of MARCH8 and SAMHD1 in macrophages**. MARCH8 reduces the virion incorporation of Env proteins into producer cells, and SAMHD1 diminishes the amount of reverse transcription (RT) in target cells, both of which lead to minimal cell damage induced by a mild amount of HIV-1 replication, and escape from immune system. These allow macrophages to become an HIV reservoir.

## Author contributions

The author confirms being the sole contributor of this work and approved it for publication.

### Conflict of interest statement

The author declares that the research was conducted in the absence of any commercial or financial relationships that could be construed as a potential conflict of interest. The reviewer TU declared a shared affiliation, though no other collaboration, with the author MF to the handling Editor, who ensured that the process nevertheless met the standards of a fair and objective review. The reviewer SS declared a shared affiliation, though no other collaboration, with the author MF to the handling Editor, who ensured that the process nevertheless met the standards of a fair and objective review.
